# Comparison between two methods of scorpion venom milking in Morocco

**DOI:** 10.1186/1678-9199-19-5

**Published:** 2013-03-28

**Authors:** Naoual Oukkache, Fatima Chgoury, Mekki Lalaoui, Alejandro Alagón Cano, Noreddine Ghalim

**Affiliations:** 1 Laboratory of Venoms and Toxins, Pasteur Institute of Morocco, 1 Place Louis Pasteur, Casablanca 20360, Morocco; 2 Institute of Biotechnology, National Autonomous University of Mexico, Cuernavaca, Mexico

**Keywords:** Scorpion venoms, Manual stimulation, Electrical stimulation, Antivenom, Lethality

## Abstract

**Background:**

The present study compared two methods used successfully in a large-scale program for the collection of scorpion venoms, namely the milking of adult scorpions via manual and electrical stimulation.

**Results:**

Our immunobiochemical characterizations clearly demonstrate that regularly applied electrical stimulation obtains scorpion venom more easily and, most importantly, in greater quantity. Qualitatively, the electrically collected venom showed lack of hemolymph contaminants such as hemocyanin. In contrast, manual obtainment of venom subjects scorpions to maximal trauma, leading to hemocyanin secretion. Our study highlighted the importance of reducing scorpion trauma during venom milking.

**Conclusions:**

In conclusion, to produce high quality antivenom with specific antibodies, it is necessary to collect venom by the gentler electrical stimulation method.

## Background

Envenomation by scorpion stings is widespread in several countries of the world. In Morocco, the two most dangerous types, the black scorpion *(Androctonus mauretanicus mauretanicus: Amm)* and the yellow scorpion *(Buthus occitanus tenutanus: Bot)* are responsible for the majority of stings [1-4].

Scorpion venom contains many proteins, peptides and other compounds, several of which are biologically active and found to be particularly useful in physiological and pharmacological research as investigatory tools [5]. Hence, there is a strong demand to obtain venom from various species of scorpions for research purposes. A variety of techniques have been described to carry out the extraction of venom from scorpions [6,7].

Numerous methods of collecting venom from scorpions have been described. The venom can be obtained by different methods such as manual extraction, electric stimulation and maceration [8].

The choice of a venom-extraction technique is very important. Moreover, to obtain high-quality venom that does not contain hemolymph contaminants and is usable for producing antivenom with a specific neutralizing antibody, the milking method is one of the parameters that should be taken into consideration.

In the current work, scorpion venom samples obtained by two different methods (manual and electrical stimulation) were compared as to protein content, biochemical characteristics and lethality in order to establish the best methodology for milking and use as animal antigen to produce antivenom with specific neutralizing antibodies.

## Methods

### Venoms

#### Venom obtained manually

*Androctonus mauretanicus* (*Amm*) and *Buthus occitanus tunetanus* (*Bo*t) scorpion venoms were manually milked as described by Louis [9]. This method employs manual stimulations of the abdomen to release venom. The venoms of many scorpion specimens were pooled, lyophilized and stocked at −20°C until use.

#### Venom obtained electrically

*Amm* and *Bot* venoms were collected by the electrical stimulation method described by Ozkan and Filazi [10]. A series of regular currents were applied to shock the scorpion until the venom was ejected. For that purpose, we immersed the body of the scorpion in a saline solution for better electrical conduction and gave a shock with electrode. We used a simple 12-volt battery. The venom droplet was recovered in a Petri dish after which the extract was kept frozen until use. Venom was recovered using distilled water and centrifuged (10,000 g). The supernatant was lyophilized (freeze dried), and then kept at −20°C until use.

### Measurement of protein concentration

Protein concentrations were determined by absorbance measurements at 280 nm, assuming an extinction coefficient (E1%280) of 10. For each venom type, we prepared a solution of venom with a final concentration of 5 mg/mL (as determined by A_280 nm_).

### Gel electrophoresis of venoms

Electrophoretic analysis of venoms was performed on 15% polyacrylamide gel in the presence of SDS under reducing conditions. All samples were dissolved in a sample buffer (50 mM Tris–HCl, pH 6.8, 0.1 M DTT, 10% glycerol, 2% SDS, and 0.1% bromophenol blue). A constant electric current of 70 mA was applied for two hours. After migration, the gel was stained with Coomassie Brilliant Blue R250.

### Spectral analysis

The absorbance spectra of venoms were obtained by reading the optical density range between 220 and 600 nm, using a Beckman spectrophotometer.

### Determination of median lethal dose (LD_50_)

Lethal potency of venoms (in micrograms of dry weight per mouse) was determined as recommended by the World Health Organization [11]. Groups of five mice were used per venom dose; venom concentration was diluted in 150 mM NaCl and injected in final volume of 500 μL by intravenous route.

Percent mortality was recorded 24 hours after injection. The median lethal dose was determined using the Software package Prism 5 GraphPad, Inc., according to the provided algorithm. Briefly a non-linear curve fitting (variable slope) was generated using the four-parameter logistical equation; constraints were imposed on minimum (0% mortality) and maximum (100% mortality) values, and no weighing was used. The same package was used to calculate median doses (Anova). Plots were generated using KaleidaGraph 4.03 (Synergy Software).

### Immunization of horses and antivenom production

The antivenom was produced through horse immunization by manually collected *Amm* venom. Following the immunization program, horses were bled. Plasma proteins were fractionated by precipitation with ammonium sulfate and immunoglobulins enzymatically digested to produce F(ab’)_2_ fragments. The fractions containing the F(ab’)_2_ fragments were extensively dialyzed against saline solution.

### Ethics committee approval

All the procedures involving animals were in accordance with the ethical principles in animal research adopted by the World Health Organization [11].

### Western blot analysis

To investigate the antivenom cross-reactivity to venom components, polyacrylamide gel electrophoresis (12.5%), supplemented with sodium dodecyl sulfate (SDS), was used to separate venom components (1 μg of venom per well), using venoms collected by manual stimulation from *Amm* and by electric stimulation from *Amm* and *Bot*. Then, separated venom proteins were electrophoretically blotted onto a nitrocellulose membrane. Membrane was blocked with 5% skim milk-phosphate-buffered solution and incubated in the presence of polyspecific F(ab')_2_*Amm* scorpion antivenom (Pasteur Institute of Morocco; 1:200 dilution), for one hour at room temperature. After the washing step, the membrane was incubated with a horseradish peroxidase-labeled goat anti-horse IgG (Sigma-Aldrich, USA) at 1:1,000 dilution for one hour at room temperature. Immunoblotted proteins were revealed using a substrate of 0.05% 4-chloro-1-naphthol in 15% methanol, in the presence of 0.03% hydrogen peroxide.

## Results

### Venom colors

**Figure 1:**
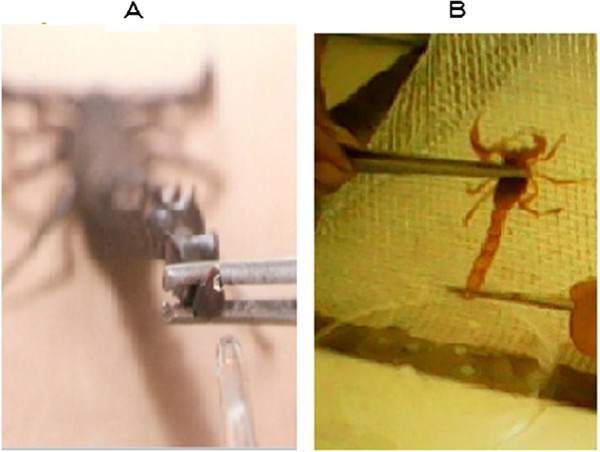
Methods of scorpion venom milking: (A) manual and (B) electrical method.

The primary characteristic associated with venom quality is the color. We compared the color of the *Amm* and *Bot* venoms obtained in both manners. Venom obtained by electrical stimulation is white and does not turn blue after milking (Figure 1). To the contrary, the manually collected venom rapidly becomes blue after milking.

### Venom biochemical characteristics

**Figure 2:**
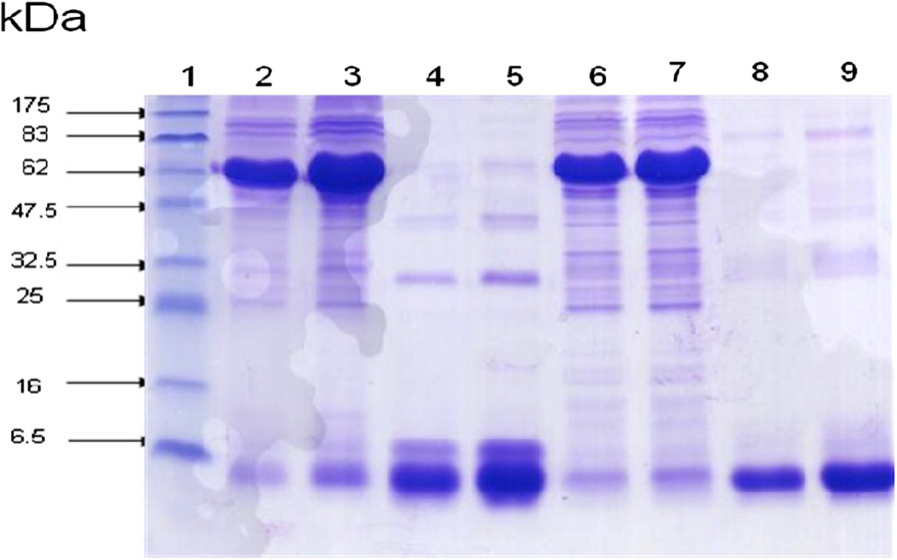
**Electrophoretic profile of venoms on polyacrylamide gel in the presence of SDS in reducing conditions.** Lane 1: molecular mass markers, lane 2: *Amm* manual method (MM) (10 μg), lane 3: *Amm* MM (20 μg), lane 4: *Amm* electric method (EM) (10 μg), lane 5: *Amm* EM (20 μg), lane 6: *Bot* MM (10 μg), lane 7: *Bo*t MM (20 μg), lane 8: Bot EM (10 μg), lane 9: *Bot* EM (20 μg).

The SDS-PAGE separation and Coommassie staining of the venom proteins were investigated. Electrophoresis analysis indicates that the venom sample obtained by electrical stimulation showed one major band of protein with molecular weights averaging 6.5 kDa (Figure 2 – lanes 2, 3, 6 and 7). Venom obtained manually showed two protein bands: the first very dense with an average molecular mass of 75 kDa corresponding to the molecular weight of hemocyanin and the second indistinct corresponding to the molecular weight of scorpion toxins (Figure 2 – lanes 4, 5, 8 and 9). Our results demonstrated that electrically collected venoms contain only proteins normally corresponding to venom toxins. However, venoms collected by manual stimulation contain a high percent of hemolymph and small amounts of toxins (Figure 2).

### Spectral analysis

The absorption spectra of proteins are currently characteristic of all scorpion venoms. We used this method to compare the two differentially milked venoms. Results show that venoms collected by the manual method have three absorption peaks: one at 280 nm and two bands were recorded near ultraviolet (220–380 nm) with a maximum at 340 nm and the other in the visible region (520–600 nm). However, venoms collected by electrical stimulation present a single absorption peak at 280 nm corresponding to the absorption wavelength of proteins (data not shown).

Indeed, protein content of venom obtained electrically was very high (absorption peak is larger compared to venom collected by manual milking). The high hemocyanin content within manually obtained venom was demonstrated by its characteristic spectral profile and absorption in ultraviolet and visible wavelengths.

### Determination of median lethal Dose (LD_50_)

Toxicity of both manually and electrically milked venoms was tested in one group of five mice and median lethal dose was determined. Results revealed respective LD_50_ values of 12.5 μg/mouse and 4.9 μg/mouse for manual and electrical milked *Amm* venom. Likewise, LD_50_s were 32.6 μg/mouse and 11 μg/mouse for manually and electrically milked *Bot* venoms (Table 1). Mice showed the same envenomation symptoms following intravenous injection of both venom samples (manual and electric milking).

**Table 1:** **Comparison of median lethal dose (LD**_**50**_**) of venom obtained manually and those obtained electrically**

**Injection way**		**LD**_**50 **_**(μg venom/mouse)**		
	*** Amm *venom**		*** Bot *venom**	
	**Manual method**	**Electric method**	**Manual method**	**Electric method**
Intravenous (μg venom/mouse)	12.5	**4.9**	32.6	**11**

The results were reported in Table 1. Median lethal dose values highlighted that toxicity was lower in the manually collected venoms than those obtained by electrical stimulation (very toxic signifies high activity). The activity of venoms collected by the electrical method was estimated to reach three fold greater values than for venoms collected manually.

### Western blot analysis

**Figure 3:**
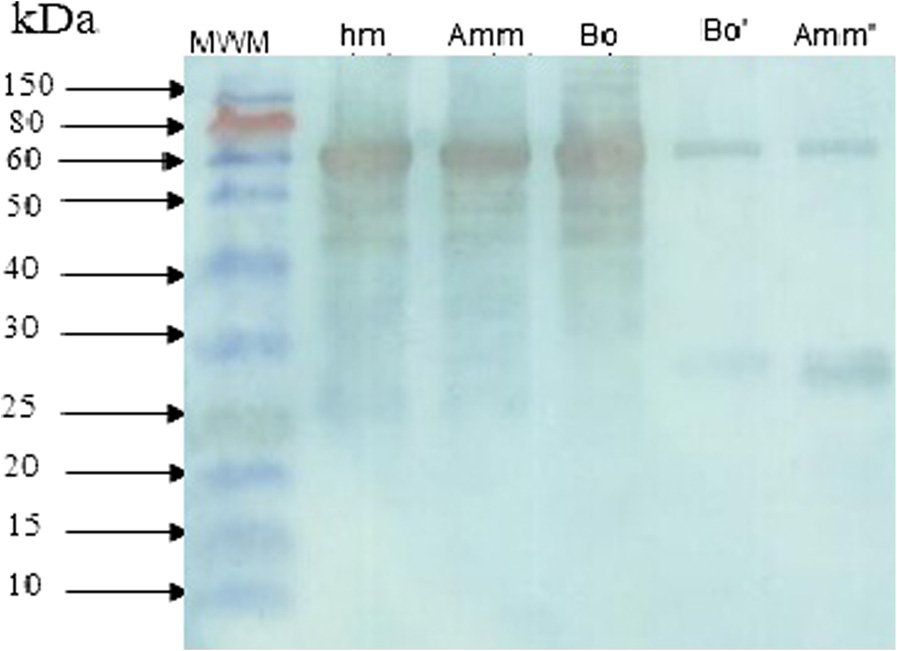
**Western blot analysis of scorpion venoms by 12.5% SDS/PAGE.** Molecular size standards (MWM) are indicated in the left (in kDa) with the corresponding venom lanes from left to right as follows: hm: hemolymph of scorpion*; Amm: Androctonus mauretanicus mauretanicus* venom collected by manual method, *Bot: Buthus occitanus tunetanus* venom collected by manual method, *Bot’: Buthus occitanus tunetanus* venom collected by electrical method and *Amm’: Androctonus mauretanicus mauretanicus* venom collected by electrical method.

*Androctonus mauretanicus mauretanicus* antivenom was produced through horse immunization with the *Amm* venom obtained by manual stimulation and subsequently used to study the immunochemical characteristics of the two differentially milked venoms. Our results showed that this antivenom strongly recognized the venom obtained by manual stimulation. Western blotting data (Figure 3) clearly revealed proteins with molecular weight of 74 kDa which are highly immunogenic.

## Discussion

Scorpion venom contains a short sequence of neurotoxin polypeptides consisting of simple, low-molecular-weight proteins that have lethal and paralytic effects. Venom toxicity varies according to several factors such as genus, species, age, physiology, feeding state and region of the scorpion. Then, major difficulties are related to standardizing venom quality [8]. To develop an antivenom that neutralizes the toxic effects of venoms as much as possible, we must have a high quality of venom with a high toxic activity (LD_50_) (containing a large amount of toxins). This activity changes according to the methodology of collecting the venom [6].

The antivenom plays an important role in the treatment of envenomation by scorpion stings. The present work found that the best antigen preparation for immunization is non-hemolymph-contaminated venom designed to produce antivenom with a high neutralizing capacity. Thus, it is important to have an effective venom-milking methodology. It is well established that the LD_50_ of scorpion venom can vary even if the venom was extracted by using a single method. For instance, Ismail *et al*. [12], found that the LD_50_ of *A*. *crassicauda* venom obtained by electric stimulation was 0.64 mg/kg, whereas Latoxan Laboratory reported an LD_50_ of 0.87 mg/kg for the same venom obtained by the same method [13]. However, Altinkurt and Altan [14] reported that the LD_50_ of *A. crassicauda* venom was 11.5 mg/kg by the maceration method. Ozkan and Filazi [10] found the toxicity of milked venom to be eight times higher than that of venom obtained by maceration of telsons.

Latifi and Tabatabai [15] reported that the mean amount of venom obtained by electric stimulation was 0.3 mg per scorpion (*A. crassicauda*), whereas 0.5 mg of venom was obtained by telson maceration.

In the present study, we found that the venom extracted by the manual method contains undesirable substances of hemolymph origin. Moreover, the venom obtained by electric stimulation is highly toxic with an LD_50_ three-fold greater than its manually collected counterpart.

Our biochemical investigations showed that venoms collected by manual stimulation have an additional band of 75 kDa that is absent in the electrophoretical profile of electrically obtained venom. Besides the spectrophotometric absorption at 280 nm (protein absorption), the absorption profiles show that the venoms obtained manually have two absorption peak regions (at 220–380 nm and 520–600 nm), which are absent in the venoms extracted by electric stimulation. According to the literature, hemocyanin is a respiratory pigment that gives scorpion hemolymph its blue color. As a percentage, it is almost the exclusive component of the hemolymph. Hemocyanin possesses a high molecular mass, and extracellular copper containing glyco-protein that displays the important function of oxygen-carrying proteins freely dissolved in the hemolymph of many mollusks and arthropods [16]. In Arthropods, hemocyanins occur mainly in the subphyla Crustacea and Chelicerata. They consist of hexameric or multihexameric complexes containing up to eight 70–75 kDa polypeptide chains with different functional and structural properties [17]. Two absorption spectra have been described, the first recorded in the near ultraviolet [265–365 nm] and the second in the visible range [400–700 nm].

The present results indicated that low-molecular-weight proteins played an important immunogenic role in the production of high-quality antivenom. Lethality and protein patterns showed variability according to the method used. Toxicity of venom obtained by the manual method was lower than when obtained by electric stimulation. Therefore, toxicity variation depends on the employed milking method, which strongly reaffirms its importance to the lethality of the venom. Venom obtained manually showed lower toxicity. Indeed, the venom obtained by manual stimulation produced similar hemolymph and Western blot profiles (similar electrophoretical feature). The most abundant protein band migrated at 75 kDa molecular weight and corresponds to hemocyanin. The large amount of hemolymph and small amounts of toxins provide the most likely explanation for its low toxicity. Moreover, the corresponding antivenom contains small amounts of specific antibodies that are capable of binding toxins and subsequently of neutralizing the lethality of the venom. This result confirms the data presented above and highlights the correlation among protein content, absorbance, toxicity and electrophoresis profile. Our result revealed that hemocyanin retrieved from the manually collected venom is a contaminant protein of hemolymph origin.

One of the interesting points is the low toxicity of the manually collected venom, a result that confirms the observations published by Inceoglu et al. [18] that scorpions, when initially stimulated, secrete a small quantity of transparent venom denominated prevenom. If secretion continues, cloudy dense venom, white in color, is subsequently released. The prevenom contains a combination of salt and several peptides that modulate ionic channels and elicit significant pain and toxicity because of a massive local depolarization. Indeed, the authors revealed that venom collected manually corresponds to the first venom type (prevenom), whereas the electrically milked venom corresponds to the second type, with high concentration of toxins.

## Conclusion

In this study, we clearly demonstrated that there is a high hemocyanin contamination in scorpion venom obtained by the manual stimulation method. Indeed, the corresponding antivenom produced from the venom obtained manually presents a high percentage of specific antibodies that neutralize the hymolymph molecules and few specific antibodies that neutralize scorpion toxins.

The venoms obtained by electrical stimulation may contribute to the production of a better antivenom with a higher neutralization potency. As to its advantages in relation to the manual method, electrical stimulation not only enables the collection of nearly 100% of the venom, but also yields more venom than manual stimulation with a higher content of toxins.

## Competing interests

The authors declare that they have no competing interest.

## Authors’ contributions

ON performed the chemical tests. CF was responsible for the venom manually obtained and its preparation. LM, director of the Pasteur Institute of Morocco, established the conditions for this study and took part by reading and correcting the manuscript. AA and ON set up the sampling technique for electrically obtained venom and contributed with technical analysis. GN provided the scorpions, supervised the venom collection and preparation, and was responsible for drafting the present manuscript and for the editorial corrections. All authors read and approved the final manuscript.
